# Retrospective Analysis of Psychological Factors in COVID-19 Outbreak Among Isolated and Quarantined Agricultural Students in a Borneo University

**DOI:** 10.3389/fpsyt.2021.558591

**Published:** 2021-04-23

**Authors:** Assikin Bin Muhamad, Nicholas Tze Ping Pang, Loganathan Salvaraji, Syed Sharizman Syed Abdul Rahim, Mohammad Saffree Jeffree, Azizan Omar

**Affiliations:** ^1^Surgical Department, Faculty of Medicine and Health Sciences, Universiti Malaysia Sabah, Kota Kinabalu, Malaysia; ^2^Community and Family Medicine Department, Faculty of Medicine and Health Sciences, Universiti Malaysia Sabah, Kota Kinabalu, Malaysia

**Keywords:** sociodemographic factors, quarantine status, indices of psychological, wellness, Borneo Agricultural Campus

## Abstract

**Introduction:** Much has been known about the psychological issues that can emerge in people who are quarantined and unable to move freely. The COVID-19 pandemic has no contrast from previous outbreaks like SARS and MERS regarding their ensuing worries and boosted anxiety levels. This article seeks to examine the unique psychological changes that occur in students who have been quarantined inside a university campus and assess sociodemographic factors associated with certain psychological factors.

**Methodology:** The data was collected from students in an Agricultural Campus. In the first phase, the factor structure of the modified National Index Psychological Wellness (NIPW) was acceptable, and to establish statistical parameters for validation an exploratory factor analysis was done. In the second phase, Independent *T*-tests, ANOVA, and Hierarchical Multiple regression were performed. Data were analyzed using the Statistical Package for the Social Sciences (SPSS) version 26.0.

**Result/Discussion:** A total of 46 male and 76 female students enrolled in this study. The Bartlett's test of sphericity was significant (*p* < 0.001) and the Kaiser–Mayer–Olkin measure of sampling adequacy for the AUDIT-M was 0.901. The Cronbach's alpha of the entire modified NIPW was 0.657 which suggests reasonable internal consistency and subscales between 0.913 and 0.924. Raw scores of 12 positive items were higher for the quarantined group except for “I can do daily routines,” “I understand what happens,” and “I understand the action that is performed is fair.” Raw mean scores of eight negative scoring items were higher in the quarantined group, except for “I feel angry” (2.88 vs. 2.89 for non-quarantined group). There were statistically significant differences between year groups for the questions “I understand what happens,” “I understand the action that is performed is fair,” and “I think everyone is good.”

**Conclusion:** Movement control orders or compulsory quarantine orders can be distressing and may cause understandable psychological sequelae. Holistic management of a quarantine center that addresses the needs and health of an individual student will give a positive impact on psychological wellness. Quarantining facilities can be a place of positivity, allowing people to live a shared experience together, provide peer support for each other, and give each other hope.

## Introduction

COVID-19, a pandemic from the coronavirus family, was first described in December 2019 in China. Malaysia had its first confirmed case on the 25th of January 2020. Subsequently, as the cases continued to rise in March 2020, Malaysia was placed under a strict nationwide movement restriction order (MRO) beginning 18 March 2020 in order to flatten the curve via state-sanctioned social distancing (1).

Hence, public university students who are studying far away from their hometowns are put in a unique quandary. The majority of them, especially those studying in Sabah and Sarawak, are a 2.5 h flight away from home. Due to flight frequencies being dramatically reduced and strict movement controls between West and East Malaysia, a lot of them have been effectively isolated in the campus (2). Furthermore, there are a small subgroup of students who have been forced to quarantine for 14 days, as they are either “persons under investigation” due to possible COVID-19 symptoms, or “persons under surveillance” due to direct or indirect contact with an individual suspected of COVID-19. In this case, quarantine involves separating groups who are potentially exposed to the disease, hence reducing the risk of infecting others. Isolation, on the other hand, is physical separation of individuals confirmed to be infected by the contagious disease (3). This is further contrasted with students who are merely subjected to the standard movement control order (MCO), who are free to go to buy food and provisions individually but otherwise cannot travel in excess of 10 km. In this case, the population of the agricultural campus was largely under MCO, with a small group subjected to quarantine, and none under isolation. This compulsory quarantine practiced in the agricultural campus is different in nature from movement restriction, as individuals under nationwide movement restrictions are still allowed to go out to purchase food and daily necessities while practicing sufficient social distancing. Quarantined students, on the other hand, were not allowed to leave their quarantine centers, and hence everything was delivered contactless to their doorsteps. The term quarantine and isolation themselves are sometimes used interchangeably, but actually carry different meanings. Both terminologies involve physical separation from the community.

There have been many literatures detailing the psychological issues that can emerge in people who are unable to move freely, or even worse, quarantined (4). However, there is still scant literature for psychological sequelae of COVID-19 quarantining and movement restrictions globally. Previous studies done with Severe Respiratory Syndrome (SRAS) and Middle East Respiratory Syndrome (MERS) survivors suggest that levels of worry and anxiety are heightened (5). As a result of quarantined cohort study with SARS survivors revealed DSM- IV psychiatric disorders was 58.9%. Furthermore, almost 25% of SARS survivors experienced Post-Traumatic Stress Disorder (PTSD) and 15.6% had significant depression (6). Older adult suicide deaths, as a proxy for diagnosable psychiatric disorders, was reportedly higher among individuals affected by SARS in 2003 and 2004 (7). There was also lower quality of life highlighted among MERS survivors that was influenced indirectly by longer duration (8). However, much of this data examined the population as a whole, instead of specifically examining the differences between the quarantined group and movement-restricted individuals.

There has been ample literature detailing the psychological issues that can emerge in people who are unable to move freely, or even worse, quarantined, including depression, insomnia, stress, anxiety, anger and fear (9). The focus on measures to prevent spread of COVID-19 may distract public attention to mental health issues, which can lead to long term health problems and even stigma if unchecked. Management of COVID-19 should hence be inclusive not only of the treatment and prevention of this pandemic, but also the mental health impact of patients and general population. One of the non-pharmacological approaches in reducing mental health issues in the population during this pandemic includes educating them to practice healthy lifestyle such as exercise. Physical activity has significant positive impacts on psychological health (10) and can enhance self-esteem, and reduce depression, anxiety, and stress.

An operational survey was performed among the students in the agricultural campus, to assess whether or not the students inside quarantine were experiencing similar, not elevated, psychological distress compared to those outside quarantine. As a result, it assessed whether those who were living outside and inside quarantine were having the same psychological experience.

The objective of this study is 2-fold. Firstly, it was to assess the levels of psychological well-being on 20 different domains. Secondly, it was to assess whether three sociodemographic factors—gender, year of study (a corollary to age), and quarantine status were associated with any differences in well-being on those domains. If the physical and psychological support for those in quarantine were similar to those outside quarantine, and there was no real deficit of experience being inside quarantine, it was then hypothesized that there should be no statistical difference in well-being, whether the individual was in or out of quarantine.

## Methodology

This was a retrospective analysis of data that was collected for operational purposes in the Agricultural Campus of a Bornean university during the beginning of the Movement Control Order in Malaysia. Hence, it was not possible to select a questionnaire that was most suited for research purposes, and the researchers did not have the opportunity to intervene in the methods of selection of respondents. Students of the agricultural campus were approached by the operational team to take part in the questionnaire and provided written informed consent for participation. The inclusion criteria were as follows:-

□ Above 18 years of age.
□ Willing to participate in the study.
□ Able to read and converse fluently in Malay language.

Apart from not consenting to join the study, there were no explicit exclusion criteria for the study, as the original data set was collected for operational, not research purposes, so no effort was made to exclude acute medical or psychiatric illness. The study participants completed two separate questionnaires: firstly, a simple demographic questionnaire containing age, gender, year of study, and whether or not the individual was quarantined or allowed to move freely; and secondly, a 20-item questionnaire adapted from the National Index of Psychological Well-being Malaysia (NIPW). Both questionnaires were in Bahasa Malaysia, the national language of Malaysia. The original NIPW is a Malay language questionnaire designed by the Public Services Department of Malaysia to assess psychological well-being for operational purposes in various governmental departments. It is used as a standard measure of well-being in University Malaysia Sabah, the site of this study. The original NIPW contains 36 questions about various aspects of wellness. For the purposes of the operational data collection, only 10 of the original questions were adopted directly, whereas eight other questions were adapted from the NIPW, and two new questions which did not measure items related to COVID-19 were added: “I feel lonely” and “I feel that the actions taken so far were reasonable.” This yielded a 20-item modified version of the NIPW. Then the questionnaire was distributed in google form, for which the students were given a link. Those answering the questionnaire were required to log in via email, and their student's registration number was then inserted. After answering the questionnaire, they were unable to repeat or modify their answers once they logged out.

Data analyses were conducted using the Statistical Package for the Social Sciences version 26.0 (SPSS, Chicago, IL) by independent researchers unrelated to the operational team to reduce bias. In the first phase of data analysis, in order to examine if the factor structure of the MODIFIED NIPW was acceptable and to establish statistical parameters for validation, an exploratory factor analysis was done. Principal component analysis with direct oblimin rotation was done to explore the factor structure of the MODIFIED NIPW. Adopting eigenvalues >1 and examining the scree plot were used to assess optimal number of factors. Examination of the pattern matrix was performed to examine respective factor loadings, and all factor loadings with correlations <0.3 were excluded. Cronbach's alpha was used to assess the internal consistency of MODIFIED NIPW and its embedded subscales. Measures of concurrent validity were unfortunately not able to be done, as this was a retrospective analysis of collected data.

In the second phase of data analysis, the data was assessed using descriptive statistics, including skewness and kurtosis to examine for assumptions of normality. Independent *T*-tests were performed to examine if there was any significant difference in scores for all 20 items of the MODIFIED NIPW for gender and quarantine status. ANOVA was performed to examine if there was any significant difference in scores for all 20 items of the year of study (divided into Year 1, Year 2, Year 3, and postgraduate). Bonferroni correction was further performed after ANOVA to assess if any significant difference remained. Correlations were calculated between all 20 items of the scale. Hierarchical multiple regressions were performed for all 20 items of the MODIFIED NIPW, adjusting hierarchically for age, gender, and quarantine status.

Permission to conduct the retrospective analysis was obtained from the Medical Ethics Committee of University Malaysia Sabah. There was no conflict of interest or sponsorship from pharmaceutical companies. However, this project was performed as part of an operational screening for UMS students, so it was impossible to ensure that all participants were blinded against each other's answers.

## Result

### Descriptive Analysis of Data

A total of 122 participants were enrolled. There are 46 male and 76 female students. Skewness and kurtosis for all variables was <2.00 suggesting a normal distribution. In terms of education levels, 100% of the respondents had completed secondary education, and there were 16 respondents that had completed first degree education.

### Factor Analysis

The Barlett's test of sphericity was significant (*p* < 0.001) and the Kaiser–Mayer–Olkin measure of sampling adequacy for the AUDIT-M was 0.901 indicating acceptable sampling (11) (**Table 2**). Principal component analysis produced three factors >1.000 when examining the eigenvalues. However, the third factor in the model had an eigenvalue barely exceeding 1.000. The second factor had an eigenvalue of 2.911, with the first two factors already accounting for 59.64% of the variance.

**Figure 1:**
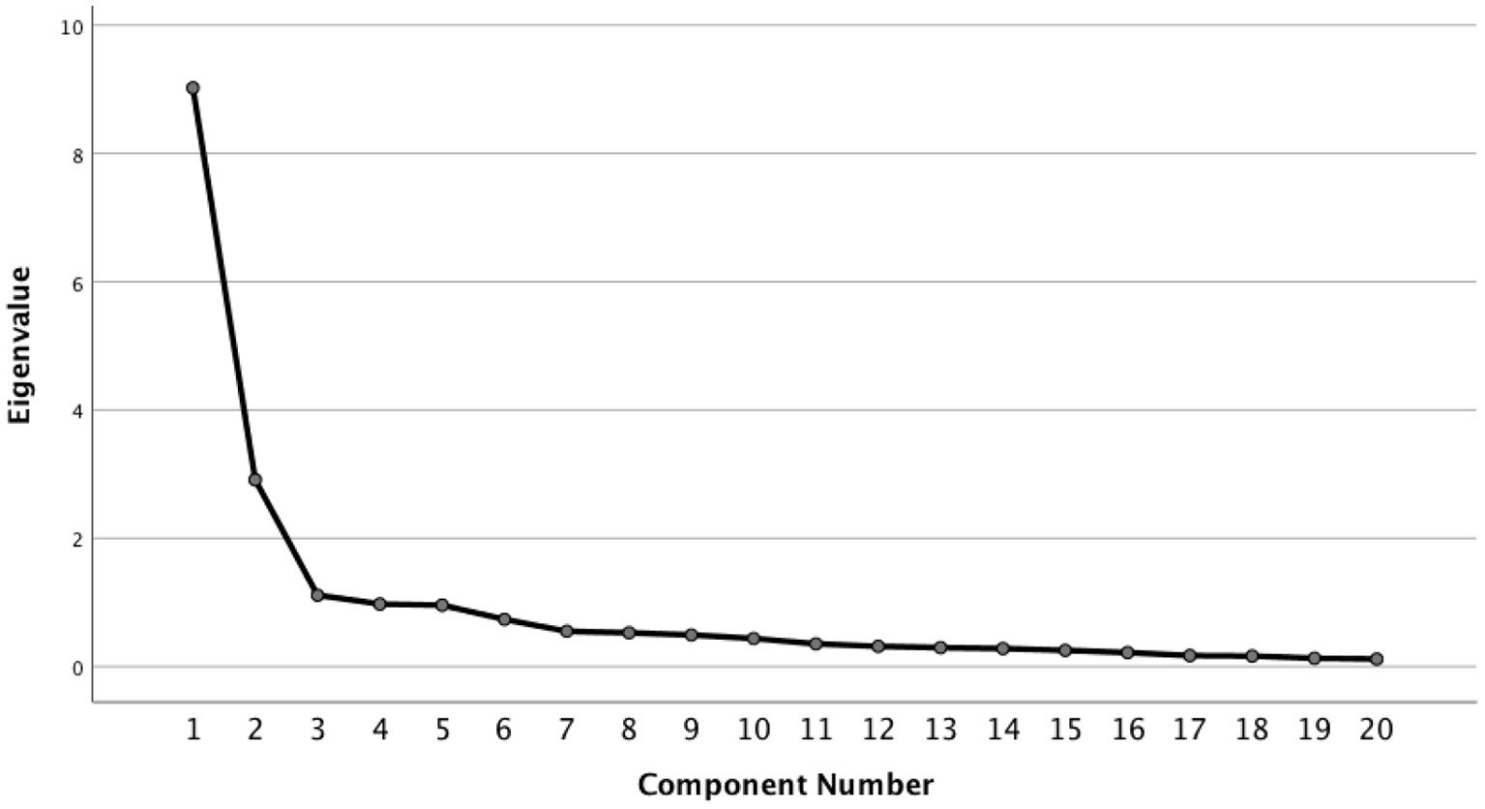
Scree plot for factor analysis of PWI-M. A two-factor appears to be suitable since it is above the kink in the plot.

When examining the scree plot (Figure 1), a two-factor appears to be suitable too, as it is above the kink in the plot. Hence correlation matrices for the two-factor solution was examined.

When a two-factor solution was used, after excluding all factors with coefficients of 0.3 and below (Table 1). The first factor, accounting for 45.116% of the variance, consisted of the 12 questions with “positive” responses, e.g., “I am happy,” and was called the “positive factor.” The correlations were all >0.458 and the Cronbach alpha for the first factor was 0.913. The second factor consisted of all the eight questions with “negative” responses, e.g., “I am angry,” and was called the “negative factor,” accounting for 14.56% of the variance. The correlations were all >0.590, with a Cronbach alpha of 0.924.

**Table 1:** Two factor solution analyze of questionnaire.

**Component**	**Total**	**Initial eigenvalues**		**Extraction sums of squared loading**			**Rotation sums of squared loadings**
		**% of variance**	**Cumulative %**	**Total**	**% of variance**	**Cumulative %**	**Total**
1	9.023	45.116	45.116	9.023	45.116	45.116	7.297
2	2.911	14.555	59.670	2.911	14.555	59.670	7.020
3	1.111	5.557	65.227				
4	0.974	4.868	70.095				
5	0.956	4.781	74.876				
6	0.736	3.678	78.5547				
7	0.550	2.749	81.303				
8	0.526	2.630	83.933				
9	0.492	2.458	86.391				
10	0.436	2.182	88.574				
11	0.353	1.767	90.340				
12	0.313	1.566	91.907				
13	0.294	1.472	93.379				
14	0.280	1.399	94.777				
15	0.251	1.256	96.033				
16	0.218	1.089	97.122				
17	0.170	0.851	97.973				
18	0.162	0.812	98.786				
19	0.128	0.638	99.424				
20	0.115	0.576	100.000				

*All factors with coefficients of 0.3 and below*.

*Extraction Method: Principal Component Analysis*.

Observing the pattern matrix for correlations (Table 2), only one item in the “Positive factor”: “I am happy” had correlations in both factors. On the other hand, also, only one item in the “Negative factor”: “I am angry” had correlations in both factors. Otherwise, none of the other 18 questions had correlations in two factors. When examining a three-factor model, five different questions had cross-correlations across three different factors, so it was less suitable as a model.

**Table 2:** Pattern matrix of questionnaire.

**No**.	**Questionnaire**	**1**	**2**
1.	I feel safe	0.676	
2.	I feel happy	0.458	−0.500
3.	I feel appreciated and protected	0.746	
4.	I feel lonely		0.819
5.	I feel negative		0.832
6.	I feel sad		0.816
7.	I feel disappointed		0.892
8.	I feel moody		0.773
9.	I'm feeling worried		0.701
10.	I'm feeling depressed		0.835
11.	I feel angry	−0.371	0.590
12	My life is very good	0.687	
13.	I can do daily routines	0.550	
14.	I'm satisfied about my life right now	0.662	
15.	I can accept it as it is	0.711	
16.	I have something important in contributing to the country	0.589	
17.	I always involve myself in the community	0.706	
18.	I understand what happens	0.791	
19.	I understand the action that is performed is fair	0.780	
20.	I think everyone is good	0.775	

*One item in the “Positive factor”: “I am happy” had correlations in both factors. One item in the “Negative factor”: “I am angry” had correlations in both factors*.

*Extraction Method: Principal Component Analysis. Rotation Method: Oblimin with Kaiser Normalization*.

*A Rotation converged in seven iterations*.

### Internal Consistency

Cronbach's alpha of the entire modified NIPW was 0.657 which suggests reasonable internal consistency. The Cronbach alpha of the subscales were between 0.913 and 0.924. Concurrent validity was not able to be performed in this study, as it was a retrospective analysis of a data set that was collected for operational purposes.

### Comparison of Means

There was no significant difference between the mean scores for all 20 questions, between quarantined and non-quarantined groups (Table 3). In the analysis of the 12 positive scoring items, the raw scores were higher for the quarantined group except for the following three items: (a) “I can do daily routines”; (b) “I understand what happens”; and (c) “I understand the action that is performed is fair.” However, there was no statistical difference between both groups as mentioned. For the eight negative scoring items, similarly, all the raw mean scores were higher in the quarantined group, except for “I feel angry” (2.88 vs. 2.89 for non-quarantined group). Again the *t*-test showed no significant difference between gender groups (Table 4).

**Table 3:** Questionnaire analysis between quarantined and non-quarantined groups.

**Items**	**Items quarantine (*n* = 16) mean (SD)**	**Non-quarantine (*n* = 106) mean (SD)**	**Mean diff. (95% CI)**	***t-*statistic (df = 120)**	***P*-value**
I feel safe	4.38 (0.806)	4.05 (1.0720)	0.328 (−0.226, 0.882)	1.172	0.244
I feel happy	2.81 (1.167)	2.79 (1.209)	0.02 (−0.619, 0.659)	0.062	0.951
I feel appreciated and protected	3.88 (1.088)	3.76 (1.1)	0.111 (−0.473, 0.694)	0.376	0.707
I feel lonely	3.69 (1.138)	3.55 (1.164)	0.14 (−0.476, 0.757)	0.451	0.653
I feel negative	3.13 (1.31)	3.02 (1.179)	0.106 (−0.529, 0.741)	0.331	0.741
I feel sad	3.31 (1.078)	3.27 (1.306)	0.039 (−0.641, 0.718)	0.113	0.91
I feel disappointed	3.13 (1.025)	3.13 (1.273)	−0.007 (−0.668, 0.654)	−0.021	0.983
I feel moody	3.19 (1.109)	3.02 (1.28)	0.169 (0.5, 0.838)	0.499	0.619
I'm feeling worried	3.56 (1.094)	3.11 (1.26)	0.449 (−0.209, 1.108)	1.351	0.179
I feel angry	2.88 (1.258)	2.89 (1.319)	−0.012 (−0.708, 0.685)	−0.034	0.973
My life is very good	3.38 (1.204)	3.31 (1.072)	0.064 (−0.515, 0.642)	0.218	0.828
I can do daily routines	2.63 (1.31)	2.69 (1.334)	−0.064 (−0.77, 0.643)	−0.178	0.859
I'm satisfied about my life right now	2.75 (1.238)	2.86 (1.245)	−0.108 (−0.769, 0.552)	−0.325	0.746
I can accept it as it is	3.38 (0.957)	3.27 (1.126)	0.101 (−0.486, 0.689)	0.342	0.733
I have something important in contributing to the country	4 (0.894)	3.49 (1.181)	0.509 (−0.101, 1.12)	1.653	0.101
I always involve myself in the community (work around it)	3.44 (0.892)	3.32 (1.109)	0.117 (−0.459, 0.692)	0.402	0.689
I understand what happens	3.88 (0.885)	4.26 (0.898)	−0.389 (−0.865, 0.087)	−1.619	0.108
I understand the action that is performed is fair	3.69 (1.138)	3.76 (1.192)	−0.077 (−0.706, 0.553)	−0.241	0.81
Performed is fair i think everyone is good	3.63 (0.957)	3.56 (1.196)	0.068 (−0.552, 0.689)	0.218	0.828

*Raw scores of 12 positive items were higher for the quarantined group except for “I can do daily routines,” “I understand what happens,” and “I understand the action that is performed is fair.” Raw mean scores of eight negative scoring items were higher in the quarantined group, except for “I feel angry”*.

**Table 4:** Questionnaire analysis between gender groups.

**Items**	**Male (*n* = 46) mean (SD)**	**Female (*n* = 76) mean (SD)**	**Mean diff. (95% CI)**	***t-*statistic (df = 120)**	***P*-value**
I feel safe	3.93	4.18 (0.976)	−0.249 (−0.635, 0.136)	−1.282	0.202
I feel happy	3.02 (1.2560)	2.66 (1.15)	0.364 (−0.076, 0.804)	1.636	0.104
I feel appreciated and protected	3.76 (0.993)	3.79 (1.158)	−0.029 (−0.435, 0.378)	−0.139	0.889
I feel lonely	3.63 (1.082)	3.53 (1.205)	0.104 (−0.325, 0.533)	0.48	0.632
I feel negative	3.02 (1.125)	3.04 (1.238)	−0.018 (−0.46, 0.425)	−0.079	0.937
I feel sad	3.22 (1.052)	3.32 (1.397)	−0.098 (−0.571, 0.375)	−0.412	0.681
I feel disappointed	3.28 (1.129)	3.04 (1.301)	0.243 (−0.215, 0.701)	1.05	0.296
I feel moody	3.11 (1.016)	3 (1.386)	0.109 (−0.357, 0.575)	0.462	0.645
I'm feeling worried	3.26 (1.021)	3.12 (1.366)	0.142 (−0.319, 0.604)	0.611	0.542
I'm feeling depressed	3.04 (1.074)	3.22 (1.312)	−0.18 (−0.635, 0.274)	−0.785	0.434
I feel angry	3 (1.174)	2.82 (1.383)	0.184 (−0.3, 0.668)	0.754	0.453
My life is very good	3.3 (1.093)	3.33 (1.088)	−0.025 (−0.428, 0.378)	−0.121	0.904
I can do daily routines	2.5 (1.329)	2.79 (1.32)	−0.289 (−0.779, 0.2)	−1.171	0.244
I'm satisfied about my life right now	2.72 (1.241)	2.92 (1.241)	−0.204 (−0.663, 0.255)	−0.878	0.381
I can accept it as it is	3.33 (1.012)	3.26 (1.159)	0.063 (−0.346, 0.472)	0.305	0.761
I have something important in contributing to the country	3.48 (1.243)	3.61 (1.108)	−0.127 (−0.556, 0.302)	−0.586	0.559
I always involve myself in the community (work around it)	3.35 (1.178)	3.33 (1.025)	0.019 (−0.382, 0.42)	0.093	0.926
I understand what happens	4.26 (0.929)	4.18 (0.89)	0.077 (−0.258, 0.411)	0.453	0.651
I understand the action that is performed is fair	3.83 (1.018)	3.71 (1.273)	0.116 (−0.322, 0.554)	0.522	0.602
I think everyone is good	3.67 (1.034)	3.5 (1.238)	0.174 (−0.257, 0.605)	0.799	0.426

*The t-test showed no significant difference between gender groups*.

As there were four different year groups, ANOVA analysis was performed (Table 5). There were statistically significant differences between year groups for three of the questions: (a) “I understand what happens”; (b) “I understand the action that is performed is fair”; and (c) “I think everyone is good.” Bonferroni correction and the differences between the groups were no longer significant except in between Post-graduate and Year 3 students for “I think everyone is good” (Table 6).

**Table 5:** Questionnaire analysis between the year groups.

**Item**	**Year**	**Mean (SD)**	**F-statistic (df = 3,118)**	***P*-value**
I feel safe	Year 1	4.31 (0.788)	1.441	0.234
	Year 2	4.1 (0.982)		
	Year 3	3.9 (1.229)		
	Postgraduate	4.57 (0.535)		
I feel happy	Year 1	2.85 (1.047)	1.288	0.282
	Year 2	3 (1.109)		
	Year 3	2.55 (1.339)		
	Postgraduate	3.14 (1.069)		
I feel appreciated and protected	Year 1	3.73 (1.079)	2.306	0.08
	Year 2	4.08 (0.888)		
	Year 3	3.51 (1.244)		
	Postgraduate	4.14 (0.69)		
I feel lonely	Year 1	1.238 (0.243)	0.235	0.872
	Year 2	1.198 (0.189)		
	Year 3	1.135 (0.162)		
	Postgraduate	0.9 (0.34)		
I feel negative	Year 1	2.92 (1.129)	0.241	0.867
	Year 2	2.98 (1.187)		
	Year 3	3.14 (1.258)		
	Postgraduate	3 (1.155)		
I feel sad	Year 1	3.62 (1.235)	1.485	0.222
	Year 2	2.98 (1.25)		
	Year 3	3.37 (1.302)		
	Postgraduate	3.14 (1.215)		
I feel disappointed	Year 1	3.23 (1.142)	0.87	0.459
	Year 2	3 (1.177)		
	Year 3	3.27 (1.303)		
	Postgraduate	2.57 (1.512)		
I feel moody	Year 1	3.23 (1.032)	0.27	0.847
	Year 2	2.95 (1.218)		
	Year 3	3.02 (1.377)		
	Postgraduate	3 (1.528)		
I'm feeling worried	Year 1	3.65 (0.892)	1.865	0.139
	Year 2	3.15 (1.292)		
	Year 3	2.96 (1.306)		
	Postgraduate	3 (1.414)		
I'm feeling depressed	Year 1	3.42 (1.137)	0.749	0.525
	Year 2	3.1 (1.194)		
	Year 3	3.12 (1.301)		
	Postgraduate	2.71 (1.254)		
I feel angry	Year 1	3.04 (1.248)	3.495	0.018
	Year 2	2.58 (1.174)		
	Year 3	3.2 (1.369)		
	Postgraduate	1.86 (1.069)		
My life is very good	Year 1	3.38 (1.134)	0.58	0.629
	Year 2	3.45 (1.011)		
	Year 3	3.16 (1.124)		
	Postgraduate	3.43 (1.134)		
I can do daily routines	Year 1	2.27 (0.962)	2.548	0.059
	Year 2	2.93 (1.403)		
	Year 3	2.57 (1.399)		
	Postgraduate	3.57 (0.976)		
I'm satisfied about my life right now	Year 1	2.62 (1.267)	1.413	0.243
	Year 2	3.1 (1.236)		
	Year 3	2.69 (1.245)		
	Postgraduate	3.29 (0.951)		
I can accept it as it is	Year 1	3.27 (1.116)	1.827	0.146
	Year 2	3.53 (0.96)		
	Year 3	3.04 (1.19)		
	Postgraduate	3.71 (0.951)		
I have something important in contributing to the country	Year 1	3.69 (0.97)	1.057	0.37
	Year 2	3.75 (1.193)		
	Year 3	3.35 (1.267)		
	Postgraduate	3.43 (0.535)		
I always involve myself in the community (work around it)	Year 1	3.23 (0.992)	0.135	0.939
	Year 2	3.33 (0.997)		
	Year 3	3.39 (1.255)		
	Postgraduate	3.43 (0.535)		
I understand what happens	Year 1	4.08 (0.977)	2.975	0.034
	Year 2	4.53 (0.679)		
	Year 3	4 (1)		
	Postgraduate	4.43 (0.535)		
I understand the action that is performed is fair	Year 1	3.69 (1.225)	2.681	0.05
	Year 2	4.08 (0.971)		
	Year 3	3.45 (1.292)		
	Postgraduate	4.29 (0.756)		
I think everyone is good	Year 1	3.54 (1.14)	4.104	0.008
	Year 2	3.83 (1.107)		
	Year 3	3.22 (1.177)		
	Postgraduate	4.57 (0.535)		

*There were statistically significant differences between year groups for questions “I understand what Happens,” “I understand the action that is performed is fair,” and “I think everyone is good”*.

**Table 6:** Questionnaire analysis between the year groups.

**Items**	**(I) Year**	**(J) Year**	**Mean difference (I-J)**	**Std. error**	**Sig**.	**95% Confidence interval**	
						**Lower bound**	**Upper bound**
		Year 2	0.208	0.262	1	−0.49	0.91
	Year 1	Year 3	0.41	0.252	0.64	−0.27	1.09
		Postgraduate	−0.264	0.442	1	−1.45	0.92
		Year 1	−0.208	0.262	1	−0.91	0.49
	Year 2	Year 3	0.202	0.221	1	−0.39	0.8
		Postgraduate	−0.471	0.426	1	−1.61	0.67
I feel safe		Year 1	−0.41	0.252	0.64	−1.09	0.27
	Year 3	Year 2	−0.202	0.221	1	−0.8	0.39
		Postgraduate	−0.673	0.42	0.668	−1.8	0.45
		Year 1	0.264	0.442	1	−0.92	1.45
	Postgraduate	Year 2	0.471	0.426	1	−0.67	1.61
		Year 3	0.673	0.42	0.668	−0.45	1.8
I feel happy		Year 2	−0.154	0.301	1	−0.96	0.65
	Year 1	Year 3	0.295	0.29	1	−0.48	1.07
		Postgraduate	−0.297	0.509	1	−1.66	1.07
		Year 1	0.154	0.301	1	−0.65	0.96
	Year 2	Year 3	0.449	0.255	0.482	−0.23	1.13
		Postgraduate	−0.143	0.489	1	−1.46	1.17
		Year 1	−0.295	0.29	1	−1.07	0.48
	Year 3	Year 2	−0.449	0.255	0.482	−1.13	0.23
		Postgraduate	−0.592	0.483	1	−1.89	0.7
	Postgraduate	Year 1	0.297	0.509	1	−1.07	1.66
		Year 2	0.143	0.489	1	−1.17	1.46
		Year 3	0.592	0.483	1	−0.7	1.89
		Year 2	−0.344	0.271	1	−1.07	0.38
	Year 1	Year 3	0.221	0.261	1	−0.48	0.92
		Postgraduate	−0.412	0.459	1	1.64	0.82
		Year 1	0.344	0.271	1	−0.38	1.07
	Year 2	Year 3	0.565	0.23	0.092	−0.05	1.18
I feel appreciated and protected		Postgraduate	−0.068	0.441	1	−1.25	1.12
		Year 1	−0.221	0.261	1	−0.92	0.48
	Year 3	Year 2	−0.565	0.23	0.092	−1.18	0.05
		Postgraduate	−0.633	0.435	0.893	−1.8	0.54
		Year 1	0.412	0.459	1	−0.82	1.64
	Postgraduate	Year 2	0.068	0.441	1	−1.12	1.25
		Year 3	0.633	0.435	0.893	−0.54	1.8
		Year 2	0.102	0.294	1	−0.69	0.89
	Year 1	Year 3	−0.015	0.283	1	−0.78	0.75
		Postgraduate	−0.28	0.497	1	−1.61	1.05
		Year 1	−0.102	0.294	1	−0.89	0.69
	Year 2	Year 3	−0.117	0.249	1	−0.78	0.55
I feel lonely		Postgraduate	−0.382	0.479	1	−1.67	0.9
		Year 1	0.015	0.283	1	−0.75	0.78
	Year 3	Year 2	0.117	0.249	1	−0.55	0.78
		Postgraduate	−0.265	0.472	1	−1.53	1
		Year 1	0.28	0.497	1	−1.05	1.61
	Postgraduate	Year 2	0.382	0.479	1	−0.9	1.67
		Year 3	0.265	0.472	1	−1	1.53
		Year 2	−0.052	0.303	1	−0.87	0.76
	Year 1	Year 3	−0.22	0.292	1	−1	0.56
		Postgraduate	−0.077	0.512	1	−1.45	1.3
		Year 1	0.052	0.303	1	−0.76	0.87
	Year 2	Year 3	−0.168	0.256	1	−0.86	0.52
I feel negative		Postgraduate	−0.025	0.493	1	−1.35	1.3
		Year 1	0.22	0.292	1	−0.56	1
	Year 3	Year 2	0.168	0.256	1	−0.52	0.86
		Postgraduate	0.143	0.486	1	−1.16	1.45
		Year 1	0.077	0.512	1	−1.3	1.45
	Postgraduate	Year 2	0.025	0.493	1	−1.3	1.35
		Year 3	−0.143	0.486	1	−1.45	1.16
		Year 2	0.64	0.319	0.282	−0.22	1.5
	Year 1	Year 3	0.248	0.307	1	−0.58	1.07
		Postgraduate	0.473	0.539	1	−0.98	1.92
I feel sad		Year 1	−0.64	0.319	0.282	−1.5	0.22
	Year 2	Year 3	−0.392	0.27	0.893	−1.12	0.33
		Postgraduate	−0.168	0.519	1	−1.56	1.22
		Year 1	−0.248	0.307	1	−1.07	0.58
	Year 3	Year 2	0.392	0.27	0.893	−0.33	1.12
		Postgraduate	0.224	0.512	1	−1.15	1.6
		Year 1	−0.473	0.539	1	−1.92	0.98
	Postgraduate	Year 2	0.168	0.519	1	−1.22	1.56
		Year 3	−0.224	0.512	1	−1.6	1.15
		Year 2	0.231	0.313	1	−0.61	1.07
	Year 1	Year 3	−0.035	0.301	1	−0.84	0.77
		Postgraduate	0.659	0.529	1	−0.76	2.08
		Year 1	−0.231	0.313	1	−1.07	0.61
	Year 2	Year 3	−0.265	0.265	1	−0.98	0.44
		Postgraduate	0.429	0.509	1	−0.94	1.79
		Year 1	0.035	0.301	1	−0.77	0.84
	Year 3	Year 2	0.265	0.265	1	−0.44	0.98
		Postgraduate	0.694	0.502	1	−0.65	2.04
		Year 1	−0.659	0.529	1	−2.08	0.76
	Postgraduate	Year 2	−0.429	0.509	1	−1.79	0.94
		Year 3	−0.694	0.502	1	−2.04	0.65
		Year 2	0.281	0.319	1	−0.58	1.14
	Year 1	Year 3	0.21	0.307	1	−0.61	1.04
		Postgraduate	0.231	0.54	1	−1.22	1.68
		Year 1	−0.281	0.319	1	−1.14	0.58
	Year 2	Year 3	−0.07	0.27	1	−0.8	0.65
		Postgraduate	−0.05	0.519	1	−1.44	1.34
		Year 1	−0.21	0.307	1	−1.04	0.61
	Year 3	Year 2	0.07	0.27	1	−0.65	0.8
		Postgraduate	0.02	0.512	1	1.35	1.39
		Year 1	−0.231	0.54	1	−1.68	1.22
	Postgraduate	Year 2	0.05	0.519	1	−1.34	1.44
		Year 3	−0.02	0.512	1	−1.39	1.35
		Year 2	0.504	0.31	0.642	−0.33	1.34
	Year 1	Year 3	0.695	0.299	0.131	−0.11	1.5
		Postgraduate	0.654	0.524	1	−0.75	2.06
		Year 1	−0.504	0.31	0.642	−1.34	0.33
	Year 2	Year 3	0.191	0.262	1	−0.51	0.89
		Postgraduate	0.15	0.504	1	1.2	1.5
		Year 1	−0.695	0.299	0.131	−1.5	0.11
	Year 3	Year 2	−0.191	0.262	1	−0.89	0.51
		Postgraduate	−0.041	0.498	1	−1.38	1.29
		Year 1	−0.654	0.524	1	−2.06	0.75
	Postgraduate	Year 2	−0.15	0.504	1	−1.5	1.2
		Year 3	0.041	0.498	1	−1.29	1.38
I'm feeling depressed	Year 1	Year 2	0.323	0.31	1	−0.51	1.15
		Year 3	0.301	0.299	1	−0.5	1.1
		Postgraduate	0.709	0.524	1	−0.7	2.11
		Year 1	−0.323	0.31	1	−1.15	0.51
	Year 2	Year 3	−0.022	0.262	1	−0.73	0.68
		Postgraduate	0.386	0.504	1	−0.97	1.74
		Year 1	−0.301	0.299	1	−1.1	0.5
	Year 3	Year 2	0.022	0.262	1	−0.68	0.73
		Postgraduate	0.408	0.497	1	0.93	1.74
		Year 1	−0.709	0.524	1	−2.11	0.7
	Postgraduate	Year 2	−0.386	0.504	1	−1.74	0.97
		Year 3	−0.408	0.497	1	−1.74	0.93
		Year 2	0.463	0.319	0.896	−0.39	1.32
	Year 1	Year 3	−0.166	0.308	1	−0.99	0.66
		Postgraduate	1.181	0.54	0.183	−0.27	2.63
		Year 1	−0.463	0.319	0.896	−1.32	0.39
	Year 2	Year 3	−0.629	0.27	0.129	−1.35	0.1
I feel angry		Postgraduate	0.718	0.519	1	−0.68	2.11
		Year 1	0.166	0.308	1	−0.66	0.99
	Year 3	Year 2	0.629	0.27	0.129	−0.1	1.35
		Postgraduate	1.347	0.512	0.058	−0.03	2.72
		Year 1	−1.181	0.54	0.183	−2.63	0.27
	Postgraduate	Year 2	−0.718	0.519	1	−2.11	0.68
		Year 3	−1.347	0.512	0.058	−2.72	0.03
		Year 2	−0.065	0.275	1	−0.8	0.67
	Year 1	Year 3	0.221	0.265	1	−0.49	0.93
		Postgraduate	−0.044	0.465	1	−1.29	1.2
		Year 1	0.065	0.275	1	−0.67	0.8
	Year 2	Year 3	0.287	0.232	1	−0.34	0.91
My life is very good		Postgraduate	0.021	0.447	1	−1.18	1.22
		Year 1	−0.221	0.265	1	−0.93	0.49
	Year 3	Year 2	−0.287	0.232	1	−0.91	0.34
		Postgraduate	−0.265	0.441	1	−1.45	0.92
		Year 1	0.044	0.465	1	−1.2	1.29
	Postgraduate	Year 2	−0.021	0.447	1	−1.22	1.18
		Year 3	0.265	0.441	1	−0.92	1.45
I can do daily routines		Year 2	−0.656	0.328	0.286	−1.53	0.22
	Year 1	Year 3	−0.302	0.316	1	1.15	0.54
		Postgraduate	−1.302	0.554	0.122	−2.79	0.18
		Year 1	0.656	0.328	0.286	−0.22	1.53
	Year 2	Year 3	0.354	0.277	1	−0.39	1.1
		Postgraduate	−0.646	0.533	1	−2.08	0.78
	Year 3	Year 1	0.302	0.316	1	−0.54	1.15
		Year 2	−0.354	0.277	1	−1.1	0.39
		Postgraduate	−1	0.526	0.357	−2.41	0.41
		Year 1	1.302	0.554	0.122	−0.18	2.79
	Postgraduate	Year 2	0.646	0.533	1	−0.78	2.08
		Year 3	1	0.526	0.357	−0.41	2.41
		Year 2	−0.485	0.311	0.73	−1.32	0.35
	Year 1	Year 3	−0.078	0.299	1	−0.88	0.72
		Postgraduate	−0.67	0.525	1	2.08	0.74
		Year 1	0.485	0.311	0.73	−0.35	1.32
I'm satisfied about my life right now	Year 2	Year 3	0.406	0.263	0.75	−0.3	1.11
		Postgraduate	−0.186	0.505	1	−1.54	1.17
		Year 1	0.078	0.299	1	−0.72	0.88
	Year 3	Year 2	−0.406	0.263	0.75	−1.11	0.3
		Postgraduate	−0.592	0.498	1	−1.93	0.75
		Year 1	0.67	0.525	1	0.74	2.08
	Postgraduate	Year 2	0.186	0.505	1	1.54	1.54
		Year 3	0.592	0.498	1	−0.75	1.93
		Year 2	−0.256	0.275	1	−0.99	0.48
	Year 1	Year 3	0.228	0.265	1	−0.48	0.94
		Postgraduate	−0.445	0.465	1	−1.69	0.8
		Year 1	0.256	0.275	1	−0.48	0.99
	Year 2	Year 3	0.484	0.232	0.237	−0.14	1.11
I can accept it as it is		Postgraduate	−0.189	0.447	1	−1.39	1.01
		Year 1	−0.228	0.265	1	−0.94	0.48
	Year 3	Year 2	−0.484	0.232	0.237	−1.11	0.14
		Postgraduate	−0.673	0.441	0.775	−1.86	0.51
		Year 1	0.445	0.465	1	−0.8	1.69
	Postgraduate	Year 2	0.189	0.447	1	−1.01	1.39
		Year 3	0.673	0.441	0.775	−0.51	1.86
		Year 2	−0.058	0.291	1	−0.84	0.72
	Year 1	Year 3	0.345	0.281	1	−0.41	1.1
		Postgraduate	0.264	0.493	1	−1.06	1.59
		Year 1	0.058	0.291	1	−0.72	0.84
I have something important in contributing to the country	Year 2	Year 3	0.403	0.246	0.628	−0.26	1.06
		Postgraduate	0.321	0.474	1	−0.95	1.59
		Year 1	−0.345	0.281	1	−1.1	0.41
	Year 3	Year 2	−0.403	0.246	0.628	−1.06	0.26
		Postgraduate	−0.082	0.467	1	−1.34	1.17
		Year 1	−0.264	0.493	1	−1.59	1.06
	Postgraduate	Year 2	−0.321	0.474	1	−1.59	0.95
		Year 3	0.082	0.467	1	−1.17	1.34
		Year 2	−0.094	0.275	1	−0.83	0.64
I always involve myself in the community (work around it)	Year 1	Year 3	−0.157	0.265	1	−0.87	0.55
		Postgraduate	−0.198	0.465	1	−1.45	1.05
		Year 1	0.094	0.275	1	−0.64	0.83
	Year 2	Year 3	−0.063	0.233	1	−0.69	0.56
		Postgraduate	−0.104	0.447	1	−1.3	1.1
		Year 1	0.157	0.265	1	−0.55	0.87
	Year 3	Year 2	0.063	0.233	1	−0.56	0.69
		Postgraduate	−0.041	0.441	1	−1.22	1.14
		Year 1	0.198	0.465	1	−1.05	1.45
	Postgraduate	Year 2	0.104	0.447	1	−1.1	1.3
		Year 3	0.041	0.441	1	−1.14	1.22
		Year 2	−0.448	0.222	0.274	−1.04	0.15
	Year 1	Year 3	0.077	0.214	1	−0.5	0.65
		Postgraduate	−0.352	0.375	1	−1.36	0.65
		Year 1	0.448	0.222	0.274	−0.15	1.04
	Year 2	Year 3	0.525^*^	0.188	0.036	0.02	1.03
I understand what happens		Postgraduate	0.096	0.361	1	−0.87	1.06
		Year 1	−0.077	0.214	1	−0.65	0.5
	Year 3	Year 2	−0.525^*^	0.188	0.036	−1.03	−0.02
		Postgraduate	−0.429	0.356	1	−1.38	0.53
		Year 1	0.352	0.375	1	−0.65	1.36
	Postgraduate	Year 2	−0.096	0.361	1	−1.06	0.87
		Year 3	0.429	0.356	1	−0.53	1.38
		Year 2	−0.383	0.291	1	−1.16	0.4
	Year 1	Year 3	0.243	0.281	1	−0.51	1
		Postgraduate	−0.593	0.493	1	−1.92	0.73
		Year 1	0.383	0.291	1	−0.4	1.16
I understand the action that is performed is fair	Year 2	Year 3	0.626	0.246	0.074	−0.04	1.29
		Postgraduate	−0.211	0.474	1	−1.48	1.06
		Year 1	−0.243	0.281	1	−1	0.51
	Year 3	Year 2	−0.626	0.246	0.074	−1.29	0.04
		Postgraduate	−0.837	0.467	0.456	−2.09	0.42
		Year 1	0.593	0.493	1	−0.73	1.92
	Postgraduate	Year 2	0.211	0.474	1	−1.06	1.48
		Year 3	0.837	0.467	0.456	−0.42	2.09
		Year 2	−0.287	0.283	1	−1.04	0.47
I think everyone is good	Year 1	Year 3	0.314	0.272	1	−0.42	1.04
		Postgraduate	−1.033	0.478	0.196	−2.31	0.25
		Year 1	0.287	0.283	1	−0.47	1.04
	Year 2	Year 3	0.601	0.239	0.08	−0.04	1.24
		Postgraduate	−0.746	0.46	0.642	−1.98	0.49
		Year 1	−0.314	0.272	1	−1.04	0.42
	Year 3	Year 2	−0.601	0.239	0.08	−1.24	0.04
		Postgraduate	−1.347^*^	0.453	0.022	−2.56	−0.13
	Postgraduate	Year 1	1.033	0.478	0.196	−0.25	2.31
		Year 2	0.746	0.46	0.642	−0.49	1.98
		Year 3	1.347^*^	0.453	0.022	0.13	2.56

*Bonferroni correction and the differences between the groups were no longer significant except in between Post-graduate and Year 3 students for “I think everyone is good”*.

* *The mean difference is significant at the 0.05 level*.

### Multiple Regression

No significant difference was encountered after hierarchical multiple regression.

## Discussion

One of the main purposes of performing the initial operational study was to assess whether the efforts to provide a pleasant quarantine experience, both infrastructural and psychologically, were sufficient. From the results, it appears that there is no statistically significant difference in all psychological indices measured between quarantined and non-quarantined groups. Pandemics are known to affect more than the physical health of the population; there are also mental health sequelae secondary to poor physical health. Therefore, it is important to maintain good physical health. Before COVID-19, individuals can focus freely on their exercise and physical activity to maintain healthy lifestyles. However, during the Covid-19 pandemic, this is significantly reduced especially for those with underlying chronic illness (12). The total energy expenditure and physical activity are significantly reduced may be due to containment and quarantine. This is due to lack of equipment, lack of large spaces, and absence of personal trainers. This in turn may cause short- and long-term health issues especially involving cardiorespiratory health and may contribute to difficulties coping with stress and anxiety during the COVID-19 pandemic (3, 10, 13). Effective strategies on promoting physical activity and exercise, hence, should be considered and implemented by either developing new or utilizing and modifying existing programs with reference to particular standard operating procedures (SOP).

It is prudent for health authorities to ensure adequate, clear-cut, and strong bases for quarantine (14). Many factors, however, determine individuals' compliance to quarantine measures. These factors are largely classified into duration of quarantine and motivation to comply (15). In this sense, two questions in this operational study actually measure the latter: “I have something important in contributing to the country” and “I always involve myself in the community.”

However, in this study, it was shown that not only did individuals comply to quarantine measures, but on raw scales of psychological wellness, their scores were statistically similar to those out of quarantine. No doubt these results could have come due to the small sample size (*n* = 14) of the quarantined group. However, as this is an analysis of an operational study, and the entire quarantined group was captured in this study, there is no justifiable ethics basis in artificially quarantining more individuals to have statistically significant results, so this is a research limitation of the study.

Looking from another angle, it is arguable that the individuals in quarantine were given an experience virtually identical to those not under quarantine. They were all housed in double- story houses with living rooms and front yards, and food, provisions and sanitary items were delivered to their doorstep on demand. Hence, physically, they may have even been more well off compared to those out of quarantine, who had to use their own money to purchase food and provisions, as they did not have any physical restrictions. Financial restrictions are a known factor for university student stress (15). Hence, this study demonstrates that it is crucial that quarantine facilities are made as indistinguishable as possible from normal life to ensure no difference, be it statistically or operationally, in psychological wellness between quarantine and non-quarantine individuals.

Psychologically, there is of course anxiety from being under investigation due to contact with COVID-19 individuals, and quarantine measures may actually have alleviated their anxiety more than standard home quarantine outside. They may also be afraid of being infected during the quarantine period, which is possible (16). In crisis, isolation is a big factor for individuals to psychologically decompensate. Conversely, in the agricultural campus, the students who were quarantined were allowed to live together in reasonably luxurious facilities. This would have provided the necessary peer support, sharing of lived experience as COVID-19 people under surveillance or investigation, which they could not have had face to face if they had been home quarantined in their respective rooms at home. Hence there is actually a sense of collective support that came out of being lodged in the quarantine center, which would have blunted the higher levels of anxiety from being suspected of being infected with COVID-19 (17). The duration of quarantine, otherwise, will increase problems in mental health, especially Post Traumatic Stress Disorder (PTSD) (18).

In comparison with their peers outside in MCO, they would have to stay in their own rooms, and be confined to their own four walls and subsist on virtual connections to talk to other individuals, as mass gatherings were strictly prohibited during the MCO. Hence, under these circumstances of collective restriction of movement, it would stand to reason that being quarantined formally is not actually as distressing or restrictive as it would be in peacetime. Moreover, quarantined individuals were given access to tele counseling services, who adopted groups of five students for individual consultations. Hence the provision of psychological services is also very crucial to reduce psychological distress in quarantine (17).

No doubt, there are other potential more insidious sequelae of quarantine or isolation that can sometimes occur. It is human nature that individuals may develop malingering or factitious disorder to expedite escape. Factitious disorder itself is sometimes difficult to diagnosed and sometimes may co-exist with true medical problems (19). From a statistical point of view, this poses a potential major research limitation, this may overestimate the presence of psychological distress in a quarantined or movement-restricted population. However, in this operational study, the focus was on comparing levels of wellness or distress rather than measuring psychological parameters against established cut-off points, as the instrument adapted did not have validated cut-off points. Qualitatively, rumor surveillance performed by doctors on the ground suggested that there was no increase in diagnosable mental disorders, as a walk-in psychiatry service was provided and tele counseling was offered to all quarantined individuals as secondary prevention.

Malingering, however is more related to background history of mental health issues or childhood health conditions (19). The other parallel situation to quarantine is that of being imprisoned; however, it is rare for quarantined or isolated individuals to develop borderline personality traits as compared to prisoners. This difference in prison settings may be due to other associated factors such as torture, personality of residents, hygiene, conduciveness, and others.

On the other hand, if malingering of psychiatric illness is present, it needs to be interpreted and judged very meticulously. Patients' symptoms need correlation with the risk factors and other history as per the standards of that illness. These subjective symptoms then require correlation with physical signs. Then further diagnoses can be established with adjunct investigations. For example, common clinical condition such as acute appendicitis, may even be missed following poor clinical judgement and an overly high index of suspicion of factitious illness (20). A diagnosis of a true psychiatric disorder needs to be performed after thorough exclusion of organic or biological disorders, as per practice within or without times of quarantine or movement restriction.

Such prolonged quarantine can no doubt result in increased fears of Covid-19 (21, 22), and have been associated with depression, anxiety, and stress in similar populations (23–25). Hence, it is crucial that early preventative measures at the university level be undertaken to increase surveillance at the alert phase (26, 27), especially taking into account the impact of digital learning on student burnout and mental health (28). Crucially, brief psychological interventions also need to be undertaken to reduce psychological morbidity, especially in rural areas (29, 30). Digital interventions can also be employed to expedite monitoring of quarantine and streamline surveillance to reduce unnecessary healthcare worker man-hours (31). However, digital tools must be judiciously used, as there is burgeoning evidence that social media itself can contribute to misinformation which can complicate the psychological health of the young people involved in the aforementioned quarantine, underpinning the importance of accurate and correctly pitched health risk communication (32). Lastly, it is imperative that culturally sensitive tools be employed to measure psychological distress to ensure accurate capture of psychopathology (33, 34).

A major limitation of the study is that we were not able to select a specific target of respondents, but rather considered any student on campus that complied with the three generic inclusion criteria, thus curtailing the generalisability of the findings. Due to the abrupt lockdown enforced by governments without warning, we were highly limited by what kind of respondents we could recruit. Hence, we had to opportunistically employ the undergraduate students who were suddenly locked down, which gave us real time data into the negative sequel of such abrupt measures. Though we were not able to include other individuals outside the campus to create a more homogenous sample, the respondents we were able to access in this time of great chaos and uncertainty gave us a valuable snapshot of the acute psychological states of acutely quarantined students at a historical moment in the time of the pandemic.

## Conclusion

Movement control orders or compulsory quarantine orders can be distressing and may cause understandable psychological sequelae. However, times of stress can also be a period of growth, and it is incumbent upon quarantining parties to ensure that the distress caused by both physical quarantining and the psychological effect of worries and fears regarding being suspected of having COVID-19 or being in contact with someone with COVID-19 is balanced out judiciously with both reasonable and comfortable physical amenities, telecommunications support, and psychological support. This paper demonstrates that in an agricultural campus in Borneo, on gross measures of psychological wellness covering both positive and negative items, there was no statistical difference between a quarantined and non-quarantined group. This reinforces the need to quarantine judiciously and quarantine well, under luxurious and privileged conditions, with ample amenities and life necessities. When properly done, quarantining facilities can be a place of positivity, allowing people to live a shared experience together, provide peer support for each other, and give each other hope.

## Data Availability Statement

The raw data supporting the conclusions of this article will be made available by the authors, without undue reservation.

## Ethics Statement

Permission to conduct the retrospective analysis was obtained from the Medical Ethics Committee of University Malaysia Sabah. Students of the agricultural campus were approached by the operational team to take part in the questionnaire and provided written informed consent for participation.

## Author Contributions

AM: data collection and discussion. NP: materials and method and discussion. LS: abstract, introduction, and discussion. SR and AO: commenter. MJ: supervisor. All authors contributed to the article and approved the submitted version.

## Conflict of Interest

The authors declare that the research was conducted in the absence of any commercial or financial relationships that could be construed as a potential conflict of interest.
